# MAP kinase dependent cyclinE/cdk2 activity promotes DNA replication in early sea urchin embryos

**DOI:** 10.1016/j.ydbio.2009.07.043

**Published:** 2009-10-15

**Authors:** J. Kisielewska, R. Philipova, J.-Y. Huang, M. Whitaker

**Affiliations:** The Institute for Cell and Molecular Biosciences, Medical School, Faculty of Medical Sciences, Newcastle University, Framlington Place, NE2 4HH, Newcastle upon Tyne, UK

**Keywords:** CyclinE, cdk2, ERK1, DNA replication, Sea urchin

## Abstract

Sea urchins provide an excellent model for studying cell cycle control mechanisms governing DNA replication in vivo. Fertilization and cell cycle progression are tightly coordinated by Ca^2+^ signals, but the mechanisms underlying the onset of DNA replication after fertilization remain less clear. In this study we demonstrate that calcium-dependent activation of ERK1 promotes accumulation of cyclinE/cdk2 into the male and female pronucleus and entry into first S-phase. We show that cdk2 activity rises quickly after fertilization to a maximum at 4 min, corresponding in timing to the early ERK1 activity peak. Abolishing MAP kinase activity after fertilization with MEK inhibitor, U0126, substantially reduces the early peak of cdk2 activity and prevents cyclinE and cdk2 accumulation in both sperm pronucleus and zygote nucleus in vivo. Both p27^kip1^ and roscovitine, cdk2 inhibitors, prevented DNA replication suggesting cdk2 involvement in this process in sea urchin. Inhibition of cdk2 activity using p27^kip1^ had no effect on the phosphorylation of MBP by ERK, but completely abolished phosphorylation of retinoblastoma protein, a cdk2 substrate, indicating that cdk2 activity is downstream of ERK1 activation. This pattern of regulation of DNA synthesis conforms to the pattern observed in mammalian somatic cells.

## Introduction

The mitogen-activated protein kinase (MAP kinase) signalling pathway plays a key role in biological responses such as cell proliferation, differentiation or death. The Ras/Raf/MEK/ERK signaling cascade is highly conserved and exists in all eukaryotic organisms (Chambard et al., 2006). In mammalian somatic cells this pathway transmits the extracellular stimuli to the nucleus (Wilkinson and Millar, 2000), stimulates cyclinD1 expression before S-phase entry (Phillips-Mason et al., 2000) and mediates the activation of cyclin-dependent kinases (cdks). The ERK signaling pathway regulates the subcellular localization of cdk2 to the nucleus (Keenan et al., 2001) and is necessary for cdk2 activation through phosphorylation of Tyr-160 (Lents et al., 2002). Abolishing ERK activity by MEK inhibition or expression of dominant-negative ERK significantly reduces cdk2 activity in mammalian cells (Lents et al., 2002).

In mammals (Meinecke and Krischek, 2003), *C. elegans* (Church et al., 1995), nemertean worms (Stricker, 2009), frog (Nebreda and Ferby, 2000) and starfish (Fisher et al., 1998) MAP kinase activity is essential for proper maturation of oocytes. The role of MAP kinase signalling at fertilization has been described in mouse (Marangos et al., 2003), frog (Guadagno and Ferrell, 1998) and sea urchin oocytes (Chiri et al., 1998; Philipova et al., 2005a,b; Philipova and Whitaker, 1998; Zhang et al., 2006, 2005). A later increase in MAP kinase activity appears to be necessary for entry into mitosis in sea urchin embryos (Chiri et al., 1998; Philipova et al., 2005b; Zhang et al., 2006, 2005) and *Xenopus* (Guadagno and Ferrell, 1998).

Amphibian, mammalian, *C. elegans* and starfish oocytes are arrested in meiotic metaphase and a fall in MAP kinase activity at fertilization consequent on the calcium signal releases the oocyte's meiotic arrest. In marked contrast, sea urchin eggs are arrested in interphase; the fertilization calcium signal triggers resumption of the first mitotic cell cycle (Whitaker, 2006) and here the situation is less clear. Initial reports described a rapid increase in ERK1 activity after fertilization (Philipova and Whitaker, 1998) and an increase in MAPK activity during the first few minutes after insemination in sea urchin eggs using MBP as a substrate (Chiri et al., 1998). This early peak occurs within the first few minutes of the sperm entering the egg and rapidly declines after 6 min. In contrast to these initial data, Carroll et al. (2000) reported a decrease in ERK activity after fertilization and Zhang et al. (2005), reported a high level of phosphorylated ERK in unfertilized sea urchin eggs which declined rapidly after fertilization.

Further work supported the initial results (Philipova et al., 2005a,b) and support the analogy with the role of ERK1 in mammalian somatic cells in interphase (Rivard et al., 1999); the analogy is deepened by the finding that ERK1 activation is necessary for S-phase progression (Philipova et al., 2005a). These finding are consistent with the fact that the unfertilized sea urchin egg rests in interphase (Whitaker, 2006) before fertilization. If indeed the analogy holds, then it would be predicted that, as in mammalian somatic cells (Dulic et al., 1992; Hua et al., 1997; Koff et al., 1992), ERK1 activity controls S-phase progression by activating cyclin/cdk pathways.

The eukaryotic cell cycle is controlled by different cyclins and their associated kinases (Murray and Hunt, 1993). In mammalian cells, levels of cyclinE and its associated kinase, cdk2, rise in late G1/early S-phase when DNA replication is initiated (Dulic et al., 1992; Hua et al., 1997; Koff et al., 1992). In *Xenopus* embryos cyclinE is constantly present until the mid-blastula transition (MBT); nonetheless cyclinE/cdk2 kinase activity fluctuates twice per cell cycle. In sea urchins it has been shown that cyclinE and cdk2 proteins are maintained at constant levels in the oocyte and throughout the first four cell divisions (Sumerel et al., 2001). CyclinE is not degraded but is instead tightly associated with the chromosomes during mitosis (Schnackenberg and Marzluff, 2002). The same group has found that cyclinE/cdk2 accumulates in the male pronucleus in early sea urchin embryos and it was suggested to play a role in sperm chromatin remodelling and in delivering cyclinE to the zygote nucleus (Schnackenberg and Marzluff, 2002).

While the role for cdk2 in initiation of DNA replication is well established in some model systems (Blow and Dutta, 2005; DePamphilis et al., 2006; Jackson et al., 1995; Knoblich et al., 1994; Moore et al., 2002), it has been suggested that cdk2 activity is not necessary for the initiation of DNA replication in sea urchin embryos (Moreau et al., 1998; Schnackenberg et al., 2007).

Here we test directly the effect of the early ERK1 activity peak on cyclinE/cdk2 protein kinase activity in regulating S-phase onset in early sea urchin embryos. We show that cdk2 activity varies during the first cell cycle; its increase at fertilization is closely correlated with and dependent on the transient, Ca^2+^-initiated post-fertilisation ERK1 activation (Philipova and Whitaker, 1998); cyclinE/cdk2 accumulation into the male and female pronuclei is likely necessary for sperm chromatin decondensation within the newly-formed zygote nucleus; cyclinE/cdk2 activity is absolutely necessary for S-phase initiation and progression, and is under the ERK1 pathway upstream control. This is further evidence of the close analogy of ERK pathway regulation of DNA synthesis in interphase-arrested mammalian cells and sea urchin eggs.

## Materials and methods

### Materials

U0126, polyclonal anti-ERK and anti-active ERK antibodies were obtained from Promega; MBP and monoclonal anti-active ERK antibody from New England Biolabs; Hoechst 33342 dye from Sigma; Roscovitine and A23187 from Calbiochem; [^3^H]-Thymidine and [^32^P]-ATP from Amersham. All other chemicals were from Sigma or BDH, pRb, p27^kip1^ and actin antibodies were from AbCam.

### Gamete handling

All experiments involving living cells were carried out at 16 °C–17 °C. Gamete handling was as previously described (Philipova and Whitaker, 2005).

### Microinjection procedure and treatment with inhibitors

Proteins were microinjected using borosilicate glass micropipettes (Harvard Apparatus GC150F-10) manipulated with an Eppendorf micromanipulator. Injections comprised 0.1% of egg volume. Concentrations in the text are final concentrations in the egg. U0126, A23187 treatments were applied 10 to 15 min before fertilization. 10 μM–100 μM. Roscovitine was applied 30 min before fertilization.

### Hoechst 33342 dye staining in vivo

The Hoechst dye 33342 was added to embryo suspensions and aliquots were scanned at intervals of 5 or more minutes using a 405 nm laser.

### Expression constructs and purification of recombinant proteins

#### RFP-PCNA

The cDNA of RFP was kindly provided by R. Tsien. RFP was fused into pGEM-T vector using NotI and SmaI restriction sites. PCNA was obtained from a GFP-PCNA construct (originally provided by C. Cardoso) using the following primers: 5′CCCGGGGATGTTCGAGGCGCGC and 3′TACTTCTTCCCTAGAATCTTCGAA. The NLS was excised and PCNA was inserted into SmaI/SalI restriction sites in a pGEM-T vector. RFP-PCNA insert was cloned between NotI and XhoI sites of a pET32a expression vector. The protein was purified as described in Kisielewska et al. (2005).

#### GFP-cyclinE and GFP-cdk2

mGFP, pBSK cyclinE and pGEX6-cdk2 were kindly provided by J. Pines. GFP was cloned between EcoRI and XbaI into a pSKII vector. CyclinE and cdk2 were cloned into XbaI and NotI into the same vector. Both constructs (GFP-cyclinE and GFP-cdk2) were then subcloned into EcoRI/NotI restriction sites of a pET32a expression vector. BL21 cells were used for transformation. The culture was grown at 30 °C to OD_600_ 0.6, induced by 0.2 mM IPTG and incubated overnight at 20 °C. The protein purification was performed according to the manufacturer's protocol.

#### C-terminus of retinoblastoma protein

pGEX2T containing the C-terminus of Rb protein was a kind gift from P. Gordon. The plasmid was transformed into BL21; the culture was grown at 37 °C to OD_600_ 0.5 and after induction was incubated at 30 °C for 5 h. Purification was carried out according to the manufacturer's protocol for Glutathione-S-Transferase (Pharmacia).

#### p27^kip1^

Mouse pCMXp27 cDNA was a kind gift from J. Pines. p27 cDNA was subcloned into BamHI/HindIII restriction sites into pCaln expression vector using following primers: 5′ GGATCCATGTCAAACGTGAGAGTGTCT and 3′AAGCTTTTACGTCTGGCGTCGAAG. *E. coli* (Origami) were used for transformation and incubated at 28 °C; after induction (0.2 mM IPTG) at OD_600_ 0.6 they were incubated overnight at 20 °C. Protein purification was performed as described in the manufacturer's protocols.

### Measurement of [^3^H]-Thymidine Incorporation

Incorporation of [^3^H]-thymidine was measured as described previously (Philipova et al., 2005b).

### Kinase assay

Cdk2 kinase activity was measured as described (Phelps and Xiong, 1998) with modifications.

Samples of 50 μl unfertilized eggs were fertilized individually in 12-wells plates for each time point (control and U0126 inhibition); then collected and washed first with 0.5 M KCl, 2 mM EDTA and 2 mM EGTA (pH6.8 for eggs or pH7.2 for embryos), then with ice-cold homogenization buffer (50 mM Tris (pH6.8 or 7.2), 200 mM KCl, 60 mM β-glycerophosphate, 15 mM EGTA (pH 6.8 or 7.2), 10 mM MgCl_2_, 0.1% Triton X-100, 1 mM benzamidine, 10 μg/ml leupeptin, 10 μg/ml aprotonin, 10 μg/ml SBTI, 0.1 mM Na_3_VO_4_, 0.1 mM NaF, 1 mM PMSF) and frozen in liquid nitrogen. Samples were stored at − 80 °C. After defrosting and homogenization on ice the whole-cell extracts were centrifuged twice at 13,000 rpm and 2 °C, aliquoted and the protein concentrations were measured using BCA kit (Pierce). After 1:5 dilution in kinase buffer (50 mM Hepes, pH 7.0; 10 mM MgCl_2_; 5 mM MnCl_2_, 1 mM DTT) kinase assays (20 μl final volume) were carried out in ATP mix (4 μg/μl Rb-C-terminus, 0.05 μCi/μl [γ-^32^P]-ATP, 5 μM ATP for 30 min at 30 °C. The reaction was terminated by Laemmli's sample buffer. Samples were loaded on 10% gels and the dried gels were analyzed by phosphoimager.

MAPK/ERK1 kinase assays were performed according to Philipova and Whitaker (1998 and 2005) using the same cell extracts.

### Western blotting

Western blotting was performed as described earlier (Philipova and Whitaker, 2005).

### Fluorescence measurements

GFP fusion protein fluorescence was observed using a Leica confocal microscope (Leica Lasertechnic GmbH, Heilderberg, Germany; Ex 488 nm line of Argon-Krypton laser). Where DNA was stained with UV excited Hoechst 33342, labelled eggs/embryos were observed using UV laser 405 nm. For RFP fusion protein confocal images were taken using 568 nm or 543 nm of Argon-Krypton laser line and 590 nm long pass filter. For confocal images the Leica objective 40×\1.25 NA oil was used.

### Image processing and analysis

Image processing was carried out using Metamorph software to obtain the average pixel intensity of the region of interest.

**Fig. 1:**
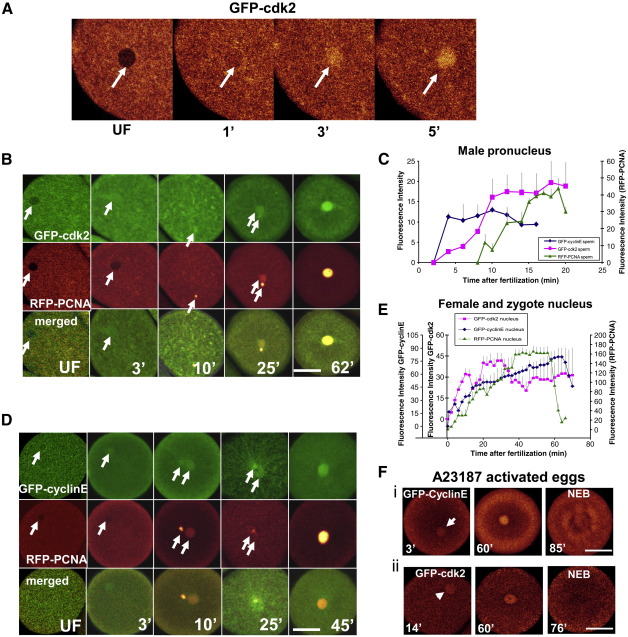
Nuclear localization of cdk2, cyclinE, and PCNA following fertilization or ionophore activation. (A) Eggs were injected with GFP-cdk2 and fertilized. GFP-cdk2 localises in the nucleus within first 3 min AF. (B, D) Eggs were co-injected with GFP-cyclinE or GFP-cdk2 together with RFP-PCNA. GFP-cdk2 and GFP-cyclinE co-localise with RFP-PCNA in fertilized eggs after 7–10 min. GFP-cyclinE and GFP-cdk2 can be detected however, in the female pronucleus within 3 min of insemination, well before pronuclear fusion. C,E. Quantified fluorescence intensity: (C) GFP-cyclinE and GFP-cdk2 co-localize with RFP-PCNA in the decondensing male pronucleus after 7–10 min; (E) parallel accumulation of GFP-cyclinE, GFP-cdk2 and RFP-PCNA in female and zygote nucleus. (F) Accumulation of GFP-cyclinE (i) and GFP-cdk2 (ii) in ionophore-activated eggs. Both proteins accumulate in the female nucleus after calcium elevation in the absence of sperm. Bar, 50 μm. Each graph represents mean and SEM of 4 individual experiments, except for GFP-cyclinE where 5 different experiments were made. For each experiment at least 3 different females were used.

Replicates are made independently in different embryos; usually at least 3 different females were used. The fluorescence intensities were normalized by division of all images by time point 0 that is equal to the fluorescence signal before fertilization except for images of Fig. 1C which include the moving sperm pronucleus. Error bars represent mean and SEM. Number of experiments are included in the figure's legend.

## Results

### CyclinE/cdk2 translocation into pronuclei precedes accumulation of PCNA

Double microinjection of GFP-cyclinE (0.1 μM) or GFP-cdk2 (0.06 μM) together with RFP-PCNA (0.06 μM) into unfertilized eggs allowed us to study their cell cycle-dependent accumulation. Fig. 1 shows the early accumulation of GFP-cdk2 (A,B) and GFP-cyclinE (D) in the female pronucleus within 3 min of fertilization. These results contrast with those of an earlier study using anti-cyclinE antibodies (Schnackenberg and Marzluff, 2002) where cyclinE was detected in the zygote nucleus only after pronuclear fusion. We confirmed however, that both GFP-cyclinE and GFP-cdk2 accumulate, albeit to different degrees, in the male pronucleus within a few min after fertilization (Fig. 1C), consistent with the previous suggestion (Schnackenberg and Marzluff, 2002) that cyclinE and cdk2 might be involved in the first step of sperm chromatin decondensation which occurs in the female cytoplasm shortly after fertilization. As we used exogenous GFP-cyclinE and GFP-cdk2 proteins and we relied on the binding of the exogenous recombinant protein to its endogenous partner (cyclinE for GFP-cdk2 and cdk2 for GFP-cyclinE) we closely monitored the cell cycle progression and observed normal cell cycle timing. Since the natural physiological state of these proteins is a stoichiometric one to one cyclinE/cdk2 complex we have additionally performed an experiment in which both recombinant cdk2 together with GFP-cyclinE were co-injected into oocytes in order to ensure the proper ratio of these proteins. The GFP-cyclinE accumulation in the nucleus that we observed was very similar to that shown in Fig. 1E and all co-injected embryos completed S-phase and entered mitosis within 70 min, the normal timing for the first cell cycle in *L. pictus* (data not shown).

Previously we and others have shown that GFP-PCNA is a sensitive and specific indicator of ongoing DNA replication (Philipova et al., 2005a; Kisielewska et al., 2005; Leonhardt et al., 2000). Here was have used an RFP variant. RFP-PCNA shows an identical pattern in its timing of nuclear accumulation to GFP-PCNA as reported previously (Kisielewska et al., 2005, Philipova et al., 2005a) (Figs. 1E and 4B).

There is no detectable nuclear RFP-PCNA accumulation in the first few minutes after fertilization (Figs. 1B, D, E). At approximately 7–10 min after sperm enters the egg RFP-PCNA is detectable in the male pronucleus, in agreement with our previous report (Philipova et al., 2005a) From this point cyclinE and cdk2 localize with PCNA in the male pronucleus until pronuclear fusion which occurs at about 25 min after fertilization (Figs. 1B, D).

### Sperm is not necessary for cyclinE/cdk2 accumulation in the female nucleus

It has been suggested that the male pronucleus is the agent that brings cyclinE/cdk2 into the zygote nucleus (Schnackenberg and Marzluff, 2002). To test whether or not the sperm nucleus is necessary for cyclinE/cdk2 nuclear uptake we used Ca^2+^ ionophore, A23187, to activate unfertilized eggs. A23187 generates a calcium elevation comparable to that during fertilization thus leading to ERK1 activation, S-phase progression and nuclear envelope breakdown (NEB) without spindle formation (Philipova and Whitaker, 1998; Whitaker and Steinhardt, 1981). We have also shown previously that ionophore-activated eggs showed GFP-PCNA accumulation in the nucleus and progress through S-phase similarly to sperm-activated eggs (Philipova et al., 2005a). Eggs injected either with GFP-cyclinE or GFP-cdk2 were activated using 20 μM A23187 (Fig. 1F). In both cases we observed early uptake of cyclinE/cdk2 into the female pronucleus and an increase in accumulation of cyclinE/cdk2 around the time of DNA replication (Fig. 1Fi, ii). This experiment clearly indicates that the input of cyclinE/cdk2 into the female and zygotic nuclei does not depend upon the presence of a sperm nucleus but rather on calcium elevation that has been shown to lead to ERK1 activation (Philipova and Whitaker, 1998).

### Cdk2 activity rises rapidly after insemination

**Fig. 2:**
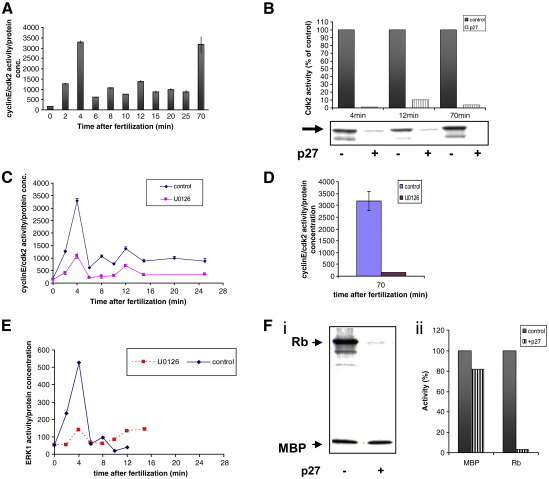
Cdk2 activity during the first zygotic cell cycle depends on ERK1 activation. (A) Cdk2 activity, measured in whole-cell extracts using C-terminus of Rb protein as substrate, increased rapidly at 4 and 70 min AF. (B) p27^kip1^ inhibits the control cdk2 peak activities at 4, 12 and 70 min AF by 99, 90 and 96.6% respectively. (C and D) 100 μM of U0126 significantly reduced the first (C, at 4 min AF) and second peak (D, at 70 min AF) of cdk2 activity. (E) Control and U0126-inhibited ERK1 activity measured in the same cell extracts. The peak corresponds in time to cdk2 early peak in activity. (F) (i) The effect of p27^kip1^ on the phosphorylation of Rb and MBP in vitro. See text for details. (ii) p27^kip1^ inhibits Rb and MBP phosphorylation by 97% and 18% respectively. All results come from 3 independent experiments. Whole-cell extracts were obtained from 3 different females.

We have measured cdk2 activity in sea urchin cell lysates (Fig. 2A). To be sure that we measure cyclinE-associated kinase activity we have monitored phosphorylation of the C-terminus of Rb (Retinoblastoma protein), known to be phosphorylated by cdk4/6 and cdk2 (Lundberg and Weinberg, 1998). It has been reported that cdk4 is not active during the first sea urchin embryonic cell cycle; its activity was first detected at 5 h after insemination (Moore et al., 2002). Initial cdk2 activity in unfertilized eggs was very low and increased rapidly by 7-fold within 2 min after fertilization (AF) reaching a peak at 4 min (19-fold over basal activity, Fig. 2A). The second similar peak occurs just before NEB at 70 min AF (Fig. 2A). The second cdk2 peak is likely to be due to the presence of cyclinA at the end of S-phase as has been previously suggested (Moreau et al., 1998). We also used p27^kip1^ recombinant protein (200 nM per reaction) to block specifically the cdk2 activity in three of the cellular extracts (at 4, 12 and 70 min. AF) showing high cdk2 activity (Fig. 2B). The activity was blocked by 90–99% (Fig. 2B). This experiment provided additional proof that the observed activity is due to cdk2. In cells treated with 100 μM of U126, a specific MEK inhibitor (Philipova et al., 2005a), cdk2 kinase activity was inhibited by 70% at 4 min and 95% at 70 min AF compared to controls (Figs. 2C, D).

MAP kinase activity measured in the same cells showed the usual post-fertilisation activation reaching its peak of activity at 4 min (Fig. 2E) with 10.2-fold increase compared to unfertilized eggs. The ERK1 activity then rapidly declined at 6 min. AF. These findings confirmed once again our previously published results (Philipova et al., 2005a; Philipova and Whitaker, 1998).

As both protein kinases, cdk2 and ERK1, reach their peaks in activities at the same time in the cell cycle, it was necessary to test whether ERK1 could contribute directly to the phosphorylation of the Rb protein. The C-terminus of Rb protein that we and others use as a substrate for cdk2 (Sumerel et al., 2001), has 8 Ser-Pro/Thr-Pro sites which are also consensus sequences for ERK phosphorylation. We used the 4 min whole-cell extract to carry out kinase assays with a mixture of both substrates, Rb and MBP, in the absence and presence of the specific cdk inhibitor, p27^kip1^ (Fig. 2Fi). Both substrates were phosphorylated in the control experiment confirming that both cdk2 and ERK are highly active at this point of the cell cycle. P27^kip1^ did not inhibit MBP phosphorylation substantially, confirming that it does not inhibit ERK1 activity. P27^kip1^ did however inhibit the phosphorylation of Rb by 97% as expected (Bastians et al., 1998; Figs. 2B, Fii), while the phosphorylation of MBP at the same time was inhibited by 18% (mean from three independent experiments). This result demonstrates that cdk2 but not ERK1 activity is responsible for the phosphorylation of the Rb protein, and also indicates either that ERK is partly inhibited by p27^kip1^, which is unlikely, or that about 18% of MBP phosphorylation is due to cdk activity as we have reported previously (Philipova and Whitaker, 1998). We conclude that cdk2 is activated immediately after fertilization and the peak in its activity corresponds closely to the sharp early peak in ERK1 activity. Our experiments with U0126-inhibited embryos indicate that cdk2 acts downstream of ERK1 in the signalling pathway.

**Fig. 3:**
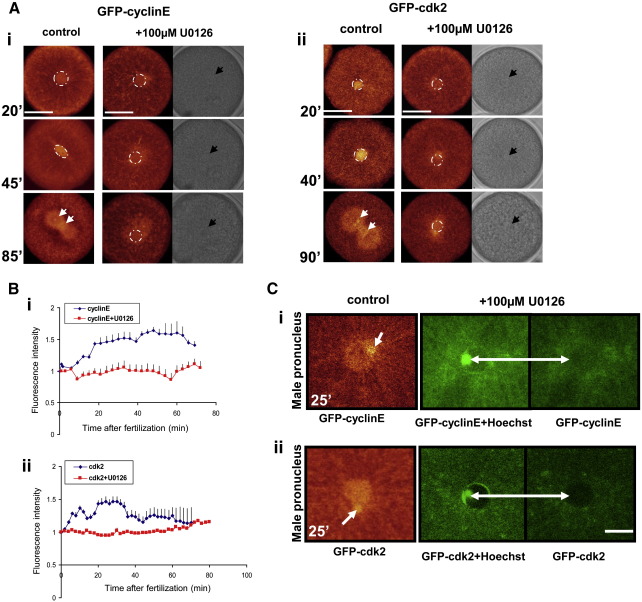
MAP kinase activation is essential for proper accumulation of cyclinE/cdk2 in male and female pronuclei, and the zygotic nucleus. (A) (i and ii) Eggs were injected with GFP-cyclinE (i) or GFP-cdk2 (ii) before fertilization. Embryos treated with MEK-specific inhibitor U0126, were unable to accumulate cyclinE or cdk2 in the zygotic nucleus when compared to control. Circles indicate the nucleus. Arrows indicate the position of centrosomes and chromosomes or nucleus when bright field was shown; bar 50 μm. The difference between images A (i) and A (ii) reflects the degree of averaging during laser scanning: in A (i) each scan was averaged times 8 and A (ii) each scan was averaged times 4. (B) (i and ii) Comparison between nuclear fluorescence intensity in control and U0126-treated embryos. (i) Embryos injected with GFP-cyclinE and (ii) GFP-cdk2. Each graph represents mean and SEM of 3 individual experiments for GFP-cdk2 and GFP-cdk2 with U1026, for GFP-cyclinE +/− U0126 5 different experiments were made. For each experiment at least 3 different females were used. (C) (i and ii) Inhibition of GFP-cyclinE (i) and GFP-cdk2 (ii) accumulation in the male pronucleus in U0126-treated embryos. Hoechst staining indicates the position of sperm chromatin after pronuclear fusion. Arrows in control eggs indicate the position of male pronucleus at pronucler fusion. Bar 10 μm.

We have shown previously that 100 μM of U0126, a specific MEK inhibitor (Gross et al., 2000; Walter et al., 2000), is sufficient to block ERK1 activity and DNA replication in sea urchin early embryos (Philipova et al., 2005a). To determine the effect of abolishing ERK activation on cyclinE/cdk2, sea urchin eggs were microinjected with GFP-cyclinE or GFP-cdk2 and treated with 100 μM of U0126 for 30 min before fertilization (Fig. 3). Control cells showed accumulation of GFP-cyclinE in male and zygote nuclei followed by its binding to chromosomes after NEB confirming earlier findings (Schnackenberg and Marzluff, 2002). In addition, we were able to detect GFP-cyclinE in centrosomes at 85 min AF (Fig. 3Ai). In contrast, in oocytes treated with MEK inhibitor, GFP-cyclinE showed significantly reduced accumulation in the nucleus (Figs. 3Ai, Bi). Similar results were obtained when cells were injected with GFP-cdk2 (Figs. 3Aii, Bii). Additionally, we have used Hoechst to determine the position of the sperm in U0126 treated eggs (Fig. 3C). There is no detectable accumulation of GFP-cyclinE and GFP-cdk2 in the male pronucleus when ERK1 activity is blocked (Fig. 3Ci and ii respectively).

### Cdk2 early activity is necessary for DNA replication and S-phase progression

**Fig. 4:**
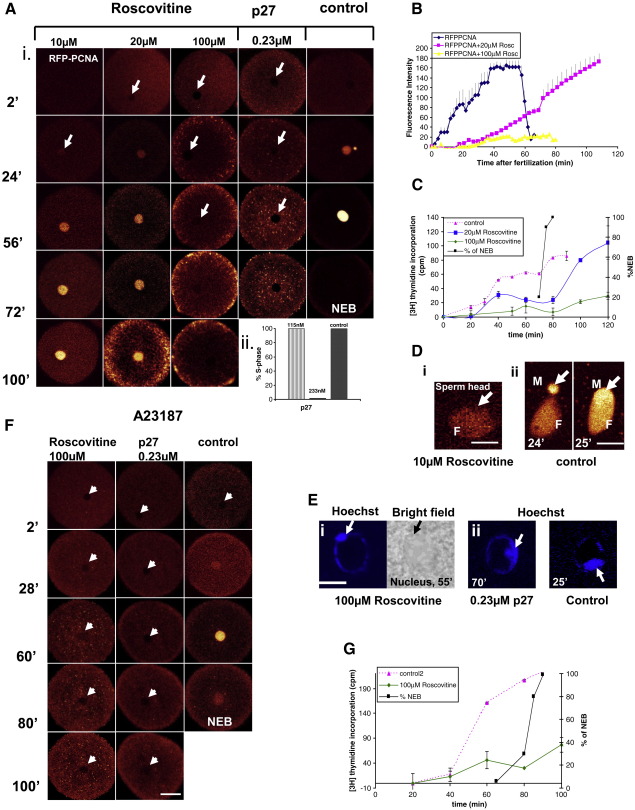
Cdk2 activity is necessary for S-phase progression. (A) (i) RFP-PCNA was co-injected with 0.23 μM p27^kip1^ or alone before fertilization. Roscovitine (10, 20, 100 μM) was added 30 min before fertilization. DNA replication was abolished by both 0.23 μM p27^kip1^ and 100 μM roscovitine. Bar 50 μm. (ii) Concentration-dependent inhibition of DNA replication by p27^kip1^. S-phase continues with lower doses of p27 but is inhibited by 0.23 μM p27. 3 different experiments for each dose were used. B. Nuclear RFP-PCNA fluorescence intensity in control embryos and in the presence of 20 or 100 μM roscovitine. Data shown as mean and SEM of 4 individual experiments. (C) [^3^H]-Thymidine incorporation in control embryos and in the presence of 20 and 100 μM roscovitine. (D) (i) Inhibition of RFP-PCNA accumulation in the male pronucleus by 10 μM roscovitine. (ii) control images of RFP-PCNA during pronuclear fusion, Bar 10 μm, M—male, F—female pronucleus. (E) Abnormal pronuclear fusion after treatment with 100 μM roscovitine (i) and lack of sperm decondensation in embryos injected with 0.23 μM p27 (ii) compared to typical pronuclear fusion at 25 min. Bar, 10 μm. (F) Lack of DNA replication (showed by RFP-PCNA incorporation into nucleus) in roscovitine-treated or p27^kip1^-co-injected ionophore-activated eggs. (G) Thymidine incorporation after ionophore alone or together with roscovitine treatment. Data in C and G shown as mean and SEM of three individual experiments. Bar, 50 μm.

Eggs were injected with RFP-PCNA and either treated with roscovitine or co-injected with 0.115–0.23 μM of p27^kip1^, a specific inhibitor of the cyclinE/cdk2 complex (see Figs. 2Fii, 4Ai). DNA replication was measured either by fluorescence intensity of RFP-PCNA (Fig. 4B) or [^3^H]-thymidine incorporation (Fig. 4C). 10, 20 and 100 μM of roscovitine were added to ASW 30 min before fertilization. 10 and 20 μM of roscovitine caused delay in DNA replication and were not sufficient to block it (Figs. 4Ai, B, C). However, the lowest concentration of the drug was enough to block the input of RFP-PCNA into the sperm head (Fig. 4Di). Cells treated with 100 μM roscovitine did not show any RFP-PCNA accumulation and S-phase progression for more than 100 min (Figs. 4Ai, B, C). All cells injected with 0.115 μM of p27 entered S-phase (Fig. 4Aii), however doubling the injection dose to 0.23 μM of p27^kip1^ completely prevented RFP-PCNA accumulation in the zygote nucleus implying a block of DNA replication (Fig. 4Ai, ii). Using Hoechst DNA dye we have noticed that there was a difference in the pattern of syngamy when cells were treated with 100 μM of roscovitine or 0.23 μM p27^kip1^ compared to controls (Fig. 4Ei, ii). Although both inhibitors prevented sperm decondensation and did not affect pronuclear fusion per se, by using higher doses of roscovitine we could observe a delay in pronuclear fusion of about 30 min together with very dense sperm heads inside the zygote nucleus suggesting lack of sperm maturation (Schnackenberg et al., 2007; Fig. 4Ei) whereas p27^kip1^ treatment showed the proper timing of syngamy (not shown), but no sperm decondensation (Fig. 4Eii). Roscovitine is a potent cdk inhibitor (Meijer et al., 1997) affecting both cdk1 and cdk2 activities. Our results are similar to those described by Tachibana et al. (2008) where it has been shown that cdk activity is necessary for the proper timing of pronuclear fusion. Inhibition of cdk2 activity by p27^kip1^ did not alter the timing of pronuclear fusion, but did prevent decondensation of the male pronucleus, implying that cdk1 controls the timing of pronuclear fusion (Tachibana et al., 2008) while cdk2 controls pronuclear decondensation. Ionophore-activated eggs were also treated with 100 μM of roscovitine or co-injected with 0.23 μM p27^kip1^. We observed an absence of RFP-PCNA accumulation in the nucleus for more than 100 min (Fig. 4F) and significantly decreased [^3^H]-thymidine incorporation in cells treated with roscovitine (Fig. 4G).

These results clearly show that cdk2 activity is necessary for sperm decondensation and DNA replication in sea urchin early embryos.

### Cdk2 activity is required for nuclear accumulation of cyclinE and cdk2

**Fig. 5:**
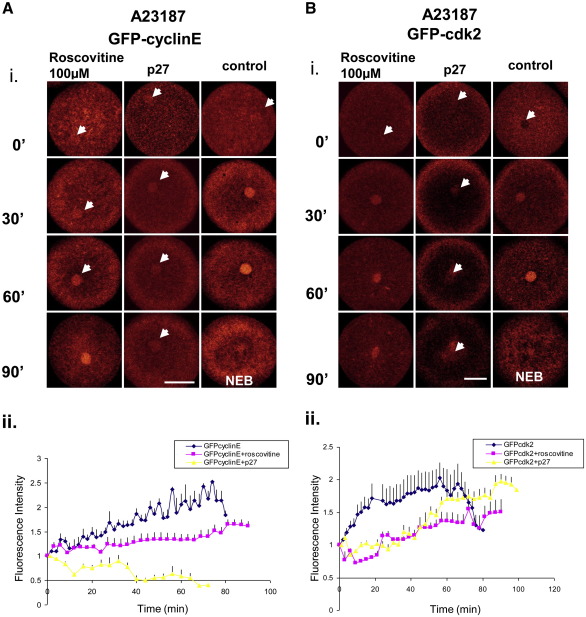
Cdk2 inhibitors affect accumulation of cyclinE and cdk2 in the female pronucleus of ionophore-activated eggs. (A and B) Representative images (i) and fluorescence intensity plots of nuclear fluorescence (ii) of GFP-cyclinE and GFP-cdk2 accumulation in the nucleus of ionophore-activated eggs after treatment with 100 μM roscovitine or injection of 0.23 μM p27. We also noted that when inhibitors were added or injected activation with ionophore took slightly longer to detect. This may be the reason why at time 0 (activation) we could detect very low fluorescence derived from GFP-cyclinE and GFP-cdk2. Roscovitine decreases the amount of cyclinE and cdk2 incorporated into nucleus. Nuclear accumulation of cyclinE when injected with p27 is almost completely abolished (Ai–ii). Cdk2 accumulation, however, while very low during first 40 min post-activation, increased rapidly after this time to about 70–80% of control (Bi–ii). Graphs represent mean and SEM of 4 individual experiments for cyclinE and *n* = 3 for cdk2 from three different females. Bar 50 μm.

To determine the behavior of cyclinE and its kinase when cdk2 activity is blocked we used ionophore-activated eggs injected with GFP tagged proteins and treated either with 100 μM roscovitine or co-injected with 0.23 μM of p27^kip1^ (Fig. 5). Both GFP-cyclinE and GFP-cdk2 accumulated to some extent in the nucleus when eggs were treated with roscovitine (Figs. 5Ai, Bi), however the fluorescence intensity was about 10–30% of controls (Figs. 5Aii, Bii). In contrast, when eggs were co-injected with 0.23 μM of p27^kip1^ which interferes with cyclinE/cdk2 complex (Russo et al., 1996), nuclear accumulation of cyclinE was almost completely abolished and fluorescence intensity decreased rapidly after 30–40 min post-activation (Fig. 5Ai, ii). Cdk2 accumulation, however, while very low during first 40 min post-activation, increased rapidly after this time to about 70–80% of control (Fig. 5Bi, ii). The difference in the pattern of fluorescence after p27 injection between cyclinE and cdk2 may be due to competition between endogenous cdk2 and exogenous GFP-cdk2 and also the appearance of cyclinA at this point of cell cycle. Two different types of cdk2 inhibition are observed here. By blocking the ATP-binding site on the kinase with roscovitine (De Azevedo et al., 1997; Meijer et al., 1997; Meijer and Raymond, 2003), cyclinE and cdk2 are still able to accumulate to some extent in the nucleus. In contrast, p27 binds to the phosphorylated cyclin/cdk complex in three places: to the peptide binding groove on the conserved cyclin box, to the N-terminal lobe of cdk2, and deeply inside the catalytic cleft of cdk, thus changing the conformation of the entire complex (Russo et al., 1996). These differences in the mode of action of the two cdk2 inhibitors are reflected in our results.

### The ERK pathway promotes DNA replication via regulation of cyclinE/cdk2 activity and translocation into male and female pronucleus

**Fig. 6:**
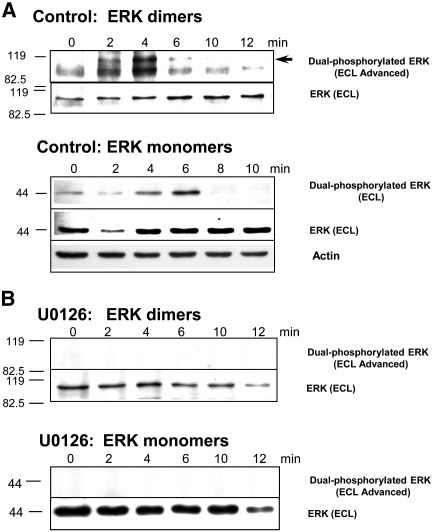
(A, B) The formation of fully activated ERK bis-phosphodimers (arrow) corresponds exactly to the measured maximum in ERK activity. Phosphorylated ERK monomers do not contribute to the extremely high ERK activity measured at 2 and especially 4 min AF. (A) Control embryos. (B) U0126 inhibition at 15 min before fertilisation. The whole-cell extracts used for measurement of ERK1 and cdk2 activities were subjected to WB using anti-dual-phosphorylated ERK (upper panels) or anti-ERK antibodies (lower panels). Equal amounts of total protein were loaded on each gel (see actin loading control). Times AF and molecular markers are shown. Note that highly sensitive ECL was used for active ERK-dimer visualisation (arrow). Non-denaturing gel was used to maintain ERK as a dimer.

Our data point clearly to a mechanism at fertilization in which transient ERK activation leads to activation and translocation of cyclinE/cdk2 into the male and female pronuclei as a prerequisite for DNA synthesis. They may appear somewhat at odds with other papers that report no major increase in ERK activity at fertilization, or even decreases (Carroll et al., 2000; Zhang et al., 2005). We have previously addressed these discrepancies by showing that the formation of fully activated dual-phosphorylated ERK1 dimers rather than activated monomers correlate with peak ERK1 activity in sea urchin zygotes (Philipova and Whitaker, 2005). In the interests of clarity and for ease of comparison with other studies, we have tested cell extracts for both dual-phosphorylated ERK dimers and monomers (Fig. 6A). As we have previously shown (Philipova and Whitaker, 2005), fully activated (Fig. 6A, arrow) dimers accumulated to maximum at 4 min AF, in strict temporal correlation with the peak of measured kinase activity (Fig. 2E). Active monomers showed an initial decrease at 2 min AF, as recorded before by others (Carroll et al., 2000; Zhang et al., 2005), followed by accumulation to a maximum at 6 min AF, after which active monomers declined, as reported by Carroll et al. (2000). These results clearly demonstrate for the first time that a maximum in activated ERK monomer (6 min AF) does not reflect a maximum in protein kinase activity (4 min AF) and also reflect the decrease in active ERK monomers at 2 and 8 min AF reported by others.

In U0126-inhibited embryos, neither active dimers nor monomers were detected, though there appeared to be a slight decrease in both inactive dimers and monomers at 12 min AF (Fig. 6B).

**Fig. 7:**
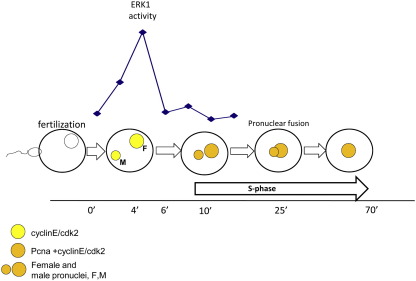
Proposed pathway controlling S-phase progression in sea urchin early embryos. Raf/MEK/ERK1 pathway controls the increase of cdk2 activity after fertilization and its translocation into male and female nucleus. At 6 min AF ERK1 activity drops down, DNA replication starts and S-phase progresses.

We have summarized our findings in Fig. 7. In sea urchin fertilization leads to calcium-dependent activation of MAPK pathway and short and rapid increase in ERK1 activity following by translocation of cyclinE/cdk2 to both male (M) and female (F) pronuclei. After ERK1 activity declined, PCNA, a marker for ongoing DNA replication, enters both pronuclei before their fusion that occurs at 25 min after fertilization. Our data lead to the conclusion that the ERK1 pathway acts upstream of cyclinE/cdk2 activity in early sea urchin embryos.

## Discussion

### Translocation of GFP-cyclinE and GFP-cdk2 into male and female pronucleus correlates with rising cdk2 activity

CyclinE/cdk2 is the main complex involved in the G1/S transition in both somatic and embryonic cells (Dulic et al., 1992; Knoblich et al., 1994; Koff et al., 1992). Here we have used fluorescently tagged human cyclinE and cdk2 to study their localization pattern in relation to MAP kinase activity. We showed previously (Philipova et al., 2005a) that in sea urchin embryos GFP-fused PCNA behaves as would be predicted of endogenous protein. Here we show that RFP-fused PCNA behaves identically to GFP-fused PCNA, demonstrating that the behaviour of the chimeric protein is that of PCNA and independent of its fluorescent tag. CyclinE localization has been studied previously using an antibody to sea urchin cyclinE (Schnackenberg and Marzluff, 2002; Sumerel et al., 2001). We have confirmed that cyclinE and its partner cdk2 localize to the male pronucleus soon after fertilization. In sea urchin, sperm chromatin goes through three steps of decondensation (Cameron and Poccia, 1994; Collas, 2000). The sperm is transformed into the male pronucleus by phosphorylation of sperm-specific histones and their exchange for cleavage-stage histones. It has been suggested that early cdk2 accumulation in the male pronucleus is associated with this process (Imschenetzky et al., 2003). We have shown previously that substantial accumulation of PCNA in the male pronucleus occurs before pronuclear fusion and this process can be blocked by aphidicolin (Philipova et al., 2005a). Here we have established the time course of accumulation of GFP-cyclinE, GFP-cdk2 and RFP-PCNA and it is clear that PCNA accumulates in the male pronucleus once its chromatin has undergone initial decondensation. This clearly indicates that DNA replication starts before pronuclear fusion in sea urchin zygotes.

Previous studies suggested that accumulation of cyclinE in the male pronucleus is the only route by which cyclinE is incorporated into the zygotic nucleus (Schnackenberg and Marzluff, 2002). In contrast, our study shows that microinjected GFP-cyclinE and GFP-cdk2 localize to the female pronucleus almost immediately after fertilization or ionophore activation. Additionally, the timing of cyclinE/cdk2 import into the female and male pronucleus correlates with the increases in ERK1 and cdk2 activity. These data demonstrate that the fertilization Ca^2+^ wave causes cyclinE/cdk2 translocation into both male and female pronuclei. It is highly likely that the difference in our and others' results are due to the different technical approaches used to detect cyclinE/cdk2. Chromatin is much more densely packed in the decondensing sperm nucleus than in the egg nucleus by a factor of two or three orders of magnitude. Since the density of cyclinE/cdk2 accumulation will reflect chromatin density, a much greater antibody or GFP signal is to be expected in the sperm pronucleus: this is indeed what we observe with the GFP reporters. This line of thought suggests that antibody detection was not sufficiently sensitive to pick up the much lower concentrations of cyclinE/cdk2 in the female pronucleus.

In *Xenopus* embryos cyclinE is constitutively present until the MBT, though cyclinE/cdk2 kinase activity fluctuates twice per cell cycle (Hartley et al., 1996). In sea urchin embryos cyclinE levels are constant until the morula stage after which they decline while cyclinE mRNA remains high until the blastula stage (Sumerel et al., 2001). Two reports have been published concerning cdk2 activity in early sea urchin embryos. The first found that cdk2 kinase activity is low and does not vary significantly from fertilization to mitosis in *S. granularis* (Moreau et al., 1998). The second showed relatively high cdk2 activity in unfertilized eggs that is maintained until 6 h after fertilization in *S. purpuratus* (Sumerel et al., 2001). Neither report sampled time points immediately after fertilization and before NEB. We used *L. pictus* lysates and the C-terminus of Rb (Lundberg and Weinberg, 1998) which provides an ideal substrate for cdk2 in the known absence of cdk4/6 activity in early embryos (Moore et al., 2002). We used p27^kip1^, a specific cyclin/cdk2 inhibitor, to block cdk2 activity (Bastians et al., 1998). We find that cdk2 activity is maintained at substantial levels throughout the cell cycle (Sumerel et al., 2001), but in addition it rises abruptly to reach a peak (19-fold over unfertilized levels) at 4 min AF, correlating with the early peak in ERK1 activity (Philipova et al., 2005a). A similar peak occurs at NEB.

### MAP kinase activation regulates cyclinE/cdk2 activity and nuclear translocation in early sea urchin embryos

MAP kinases and cdks are key enzymes that regulate cell proliferation. It is known that the principal effect of ERK in proliferating somatic cells is the direct phosphorylation of transcription factors that stimulates the expression of cyclinD/cdk4/6 (Lew, 2003). Additionally, ERK1/2 also targets the cyclin/cdk inhibitor p27^kip1^ for degradation (Wilkinson and Millar, 2000). There is no evidence in the literature of direct biochemical interactions between ERK1 and cdk2. However, it has been reported that the ERK pathway regulates cdk2 nuclear translocation (Keenan et al., 2001) via its phosphorylation on Tyr-160 (Lents et al., 2002). These reports support our current findings that demonstrate the importance of the post-fertilization ERK1 peak for cdk2 activation and cyclinE/cdk2 pronuclei translocation, and are consistent with our earlier reports that show that ERK activation is essential for S-phase onset (Philipova et al., 2005a; Philipova and Whitaker, 1998) Inhibiting MEK activity abolished both the post-fertilization and the NEB peak in ERK activity; it prevented not only sperm decondensation but also GFP-cyclinE and GFP-cdk2 accumulation in both pronuclei. These results clearly demonstrate that cyclinE/cdk2 localization and activity is under ERK pathway control. Thus, there is a strict correlation between calcium elevation, ERK1 activation, cdk2 early activity and cdk2/cyclinE accumulation in male and female pronuclei that leads to nuclear accumulation of PCNA in early sea urchin embryos.

The results presented here confirm our previous observations that ERK1 activity rises rapidly at fertilization (Philipova et al., 2005a; Philipova and Whitaker, 1998). They were supported by results of Chiri et al. (1998) who reported a 50% post-fertilization increase in MBP activity followed by another ERK peak before mitosis. In contrast, Carroll et al. (2000) showed a small, not significant increase in ERK activity at 5 min AF and decrease within the next 5 min. Later Zhang et al. (2005) reported a high level of phosphorylated ERK1 in unfertilized eggs which declined rapidly after fertilization. In these reports the authors used anti-phospho ERK antibody to detect ERK activity. We have recently reported that phosphorylated ERK1 monomers contribute very little to ERK1 activity; basal ERK1 activity is due to a semi-active ERK1 homodimer in which only one ERK1 molecule is dual-phosphorylated (Philipova and Whitaker, 2005). The fertilization calcium wave triggers phosphorylation of the second dimerised partner and the accumulation of bis-phosphodimers that are responsible for the post-fertilization peak in MAP kinase activity (Philipova and Whitaker, 2005). We report here a direct comparison between bis-phosphodimer and phosphorylated 42/44 kDa monomers and show that phosphomonomer activity (Zhang et al., 2006, 2005) does not correlate with peak ERK activity, rather that ERK activity correlates with the bisphoshorylated dimers (Philipova and Whitaker, 2005). Zhang et al. (2006) also showed that treatment of unfertilized sea urchin eggs with low doses of MEK inhibitors decreases ERK activity and drives eggs into mitosis without initiation of DNA replication. These results are consistent with our findings that inactivation of the ERK pathway prevents S-phase in fertilized eggs (Philipova et al., 2005a).

### Cdk2 activity is necessary for DNA replication in sea urchin embryos

While initiation of DNA replication is well studied in *Xenopus* cell free systems and mammalian cells (Blow and Dutta, 2005; DePamphilis et al., 2006) very little is known about this process during first and subsequent embryonic cell cycles. However, the accepted view is that promotion of metazoan DNA replication occurs upon sequential action of cdk2 and cdc7 (DePamphilis et al., 2006; Walter et al., 2000). The requirement for cyclinE/cdk2 activation for initiation of DNA replication has been shown in human cells (Krude, 2000; Machida et al., 2005) and *Xenopus* cell free system (Strausfeld et al., 1994; Walter et al., 2000). Moore et al. (2002) showed that elimination of cyclinE/cdk2 translocation into nucleus abolishes DNA replication in *Xenopus* cell extracts.

In contrast to these studies, two reports suggested that cdk2 activity is not necessary for DNA replication in early sea urchin embryos (Moreau et al., 1998; Schnackenberg et al., 2007). In the first report (Moreau et al., 1998) the authors used p21 as a cdk2 inhibitor together with less specific cdk2 inhibitors: dimethylaminopurine (DMAP) and Olomoucine. The second report (Schnackenberg et al., 2007) suggests that cyclinE/cdk2 activity is required only for sperm maturation but not DNA replication.

To characterize the cdk2 involvement in DNA replication in sea urchin early embryos in vivo we have used two potent cdk2 activity inhibitors: p27^kip1^ and roscovitine. We very deliberately did not use another cdk inhibitor — p21^Waf1/Cip1^ (p21) since it is known that the N-terminus of p21 binds and inhibits cdk/cyclin kinase activity whereas the C-terminus binds and inhibits PCNA (Gulbis et al., 1996; Rousseau et al., 1999). Thus, injection of p21 into cell will result in cell cycle arrest via binding to both cdk2 and PCNA; indeed it is surprising that ongoing DNA replication was reported in the presence of injected p21 by Moreau et al. (1998). In contrast, only a single inhibitory activity has been established for p27 which is the ability of its highly conserved N-terminus to bind and inhibit cyclin/cdk complexes (Bastians et al., 1998). To confirm our results we have used also another potent cdk2 inhibitor — roscovitine, which is known to compete with ATP in the ATP-binding pocket of cdk2 (Meijer et al., 1997; Meijer and Raymond, 2003).

We have shown a close correlation between ERK1 and cdk2 activity peaks soon after fertilization which when inhibited prevent DNA replication as measured by accumulation of RFP-PCNA. Lower doses of roscovitine substantially delay DNA replication onset and, most likely, lead to DNA damage, which can be observed by over-accumulation of PCNA in the nucleus. This effect was previously reported by Schnackenberg et al. (2007). Additionally lower doses of roscovitine stopped PCNA accumulation in the sperm head preventing sperm maturation at this stage of cell cycle (Imschenetzky et al., 2003). We were aware that 10 and 20 μM of roscovitine would likely be insufficient to block cdk2 activity fully, as 50–100 times higher concentrations are needed in *Xenopus* cell extracts (Li and Blow, 2004). In fact 100 μM of the inhibitor was sufficient in sea urchin zygotes to completely abolish DNA replication in our study. We also noted that blocking the ATP-binding site on cdk2 using roscovitine did not prevent cyclinE/cdk2 nuclear accumulation, whereas interfering with cyclinE/cdk2 complex formation by using p27 prevented it. p27 injected into cells and added to cell extracts was able to block cdk2 kinase activity and completely abolish DNA replication, most likely by inhibiting prereplication complex formation during the early minutes after fertilization.

## Conclusions

Our study clearly demonstrates that the early post-fertilization peak in ERK1 activation promotes cyclinE/cdk2 activation and their translocation into male and female pronuclei in order to promote sperm decondensation, PCNA binding, and DNA replication in early sea urchin embryos. As previously demonstrated (Philipova et al., 2005a), G1-arrested sea urchin eggs use the ERK signalling pathway very differently to oocytes arrested during meiosis, where a fall in ERK pathway activity is essential for cell cycle progression (Whitaker, 2006). Thus, in interphase-arrested sea urchin eggs, fertilization leads to activation of pathways closely analogous to those found to control the onset of S-phase in G1-arrested mammalian somatic cells with a different role for the ERK pathway than in many other oocytes even those arrested in G1 (Mori et al., 2006).
